# SARS-CoV-2 Continuous Genetic Divergence and Changes in Multiplex RT-PCR Detection Pattern on Positive Retesting Median 150 Days after Initial Infection

**DOI:** 10.3390/ijms23116254

**Published:** 2022-06-02

**Authors:** Dakai Liu, George D. Rodriguez, Hang-Yu Zhou, Ye-Xiao Cheng, Xiaofeng Li, Wenwen Tang, Nishant Prasad, Chun-Cheng Chen, Vishnu Singh, Eric Konadu, Keither K. James, Maria F. Bahamon, Yvonne Chen, Sorana Segal-Maurer, Aiping Wu, William Harry Rodgers

**Affiliations:** 1 Department of Pathology and Clinical Laboratories, NewYork-Presbyterian Queens, 56-45 Main Street Flushing, New York, NY 11355, USA; dal9165@nyp.org (D.L.); vis9079@nyp.org (V.S.); erk9029@nyp.org (E.K.); kej9047@nyp.org (K.K.J.); mab9384@nyp.org (M.F.B.); yvc9018@nyp.org (Y.C.); 2 Division of Infectious Disease, NewYork-Presbyterian Queens, 56-45 Main Street Flushing, New York, NY 11355, USA; nip9027@nyp.org (N.P.); sxsegalm@nyp.org (S.S.-M.); 3 Institute of Systems Medicine, Chinese Academy of Medical Sciences & Peking Union Medical College, Beijing 100005, China; zhy@ism.cams.cn (H.-Y.Z.); yexiaocheng9@foxmail.com (Y.-X.C.); 4 Suzhou Institute of Systems Medicine, Suzhou 215123, China; 5 National Clinical Research Center for Respiratory Disease, State Key Laboratory of Respiratory Disease, Guangzhou Medical University, Guangzhou 510182, China; xiaofeng82@hotmail.com; 6 Vascular Biology and Therapeutics Program, Department of Pharmacology, Yale University School of Medicine, New Haven, CT 06520, USA; wenwentang@gmail.com; 7 Department of Surgery, NewYork-Presbyterian Queens, 56-45 Main Street Flushing, New York, NY 11355, USA; chc4003@med.cornell.edu; 8 Department of Pathology and Laboratory Medicine, Weil Cornell Medical College, 1300 York Avenue, New York, NY 10065, USA

**Keywords:** SARS-CoV-2, COVID-19, next generation sequencing, mutation, genetic divergence, viral variants, multiplex RT-PCR

## Abstract

Being in the epicenter of the COVID-19 pandemic, our lab tested 193,054 specimens for SARS-CoV-2 RNA by diagnostic multiplex reverse transcription polymerase chain reaction (mRT-PCR) starting in March 2020, of which 17,196 specimens resulted positive. To investigate the dynamics of virus molecular evolution and epidemiology, whole genome amplification (WGA) and Next Generation Sequencing (NGS) were performed on 9516 isolates. 7586 isolates with a high quality were further analyzed for the mutation frequency and spectrum. Lastly, we evaluated the utility of the mRT-PCR detection pattern among 26 reinfected patients with repeat positive testing three months after testing negative from the initial infection. Our results show a continuation of the genetic divergence in viral genomes. Furthermore, our results indicate that independent mutations in the primer and probe regions of the nucleocapsid gene amplicon and envelope gene amplicon accumulate over time. Some of these mutations correlate with the changes of detection pattern of viral targets of mRT-PCR. Our data highlight the significance of a continuous genetic divergence on a gene amplification-based assay, the value of the mRT-PCR detection pattern for complementing the clinical diagnosis of reinfection, and the potential for WGA and NGS to identify mutation hotspots throughout the entire viral genome to optimize the design of the PCR-based gene amplification assay.

## 1. Introduction

Since the outbreak of the Coronavirus Disease 2019 (COVID-19) pandemic started in late December 2019, Severe Acute Respiratory Syndrome Coronavirus 2 (SARS-CoV-2) has caused over 500 million infections worldwide (https://covid19.who.int; accessed on 29 April 2022). SARS-CoV2 genetic diversity has primarily originated from random mutations and recombination. The high mutation rate of this RNA virus leads to abundant variations within its genome. As the virus continues to evolve, over 150,000 types of mutations have been detected in the SARS-CoV-2 genome. As a result, over 1600 lineages (variants) have emerged, including the following Variants of Concern: Alpha (B.1.1.7 and related sub-lineages) [1], Delta (B.1.617.2 and related sub-lineages) [2] and Omicron (BA.1/BA.2 and related sub-lineages) [3], etc. The analysis of the genetic divergence and monitoring of the evolutionary capacity of SARS-CoV-2 over time is not only crucial to track the phylodynamics of the pandemic pattern, but also important to understand the value of the multiplex reverse transcription polymerase chain reaction (mRT-PCR) detection patterns to complement clinical diagnoses and assist in identifying viral variants.

In clinical diagnostic virology and epidemiology, multiplex polymerase chain reaction (mPCR) and mRT-PCR have been widely used for identifying genotypes and detecting mutations. For example, Hepatitis C virus variants with a single nucleotide change or multiple nucleotide changes are well characterized by mRT-PCR [4]. The use of human papilloma virus (HPV) genotype sequence-specific PCR primers/probes allows for the amplification of the target viral gene only if the target DNA remains in the specimens. Following amplification, the detection pattern is a diagnostic tool for the presence or absence of the target gene to identify HPV genotypes [5]. Recently, we have used Hecin^R^ mRT-PCR assays to detect different SARS-CoV-2 variants (B.1.351 and B.1.1.7), which has been verified by Next Generation Sequencing. This suggests that this mRT-PCR assay is valuable in identifying SARS-CoV-2 variants. Of note, detection pattern changes of the nucleocapsid gene (*N* gene) amplicon and envelope gene (*E* gene) amplicon have been observed using mRT-PCR, though the significance and application of detection pattern change have not been evaluated. Moreover, given that whole genome amplification (WGA) is especially valuable in its ability to derive the whole genome from only the virus isolates present in the clinical specimen, WGA was employed along with Next Generation Sequencing in our study. As such, we used this approach to investigate the dynamics of virus molecular evolution, epidemiology, mutation frequency, mutation spectrum, and the impact of viral genetic divergence on gene amplification-based testing. Furthermore, we analyzed the utility of the mRT-PCR detection pattern among 26 reinfection patients.

## 2. Results

Our lab tested 193,054 specimens for SARS-CoV-2 RNA by diagnostic mRT-PCR from 6 March 2020 to 15 April 2022. 17,196 (8.9%) specimens resulted positive. These specimens were collected from 91,114 unique individuals one time or multiple times, and 12,966 of them were diagnosed with SARS-CoV-2 infection with an average incidence of 14.2%. To investigate the continuous genetic divergence of SARS-CoV-2 variants, 9516 positive isolates (See Material & Methods) were sequenced. After filtering, 7586 isolates (79.7%) were analyzed for the mutation frequency and spectrum over time. A sequence analysis revealed the continuous genetic divergence of SARS-CoV-2 variants. 

**Figure 1:**
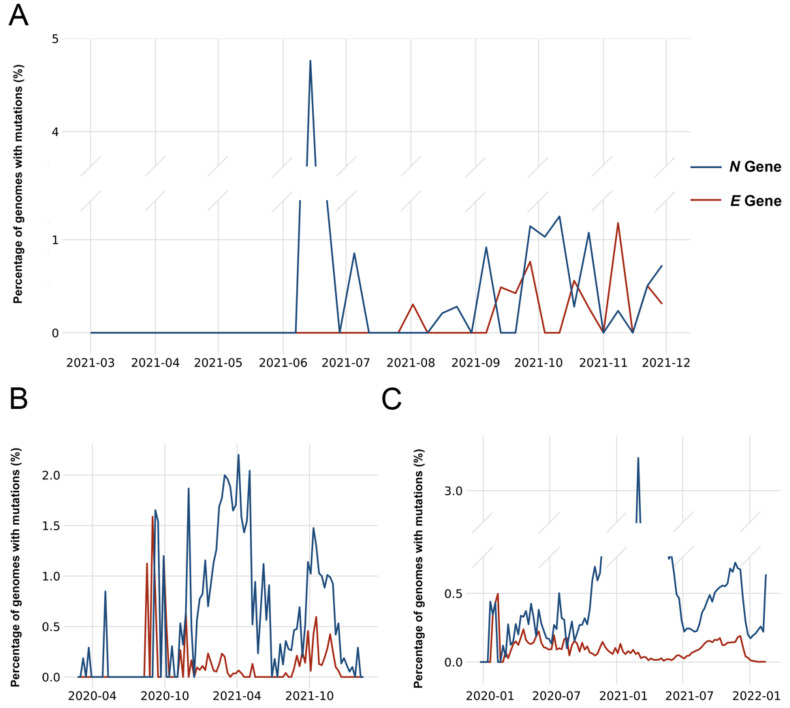
Trends of significant mutations in the genome over time. The graph shows the proportion of isolates containing significant mutations in the primers and probes of the *E* gene amplicon (red) and *N* gene amplicon (blue). ((**A**), *n* = 7586) The changes in the local New York City communities are depicted from March 2020–December 2020, ((**B**), *n* = 118,913) New York State April 2020–December 2021, and ((**C**), *n* = 7,771,134) whole world January 2020–January 2022.

It is reported that any mutations within the probe region and located up to five nucleotides from the 3′ end of primers result in a deleterious effect on PCR amplification [6,7,8,9]. We investigated these significant mutations in this study. The mutation frequency within the viral genome, as corresponding to the sequences of primers and probe, is plotted over time for the isolates collected from the local New York City communities during the period of March 2021 to December 2021 (Figure 1A).

The significant mutations continuously occurred within the regions of the *E* gene amplicon and *N* gene amplicon in the viral genome. The mutation frequency ranged from 0.5% to 1%. The mutation tended to occur in only one amplicon, although there were some rare exceptions. The mutation frequency of the *E* gene amplicon and *N* gene amplicon generally showed an indirect relationship, where high frequencies of the *N* gene amplicon occurred at the same time as lower frequencies of the *E* gene amplicon, and vice versa. Most shifts in mutation frequencies corresponded to changes of the viral variants circulated within the community as correlated with the changes of the mRT-PCR detection pattern. This trend was also observed in the whole of New York State (Figure 1B) and worldwide (Figure 1C). 

**Figure 2:**
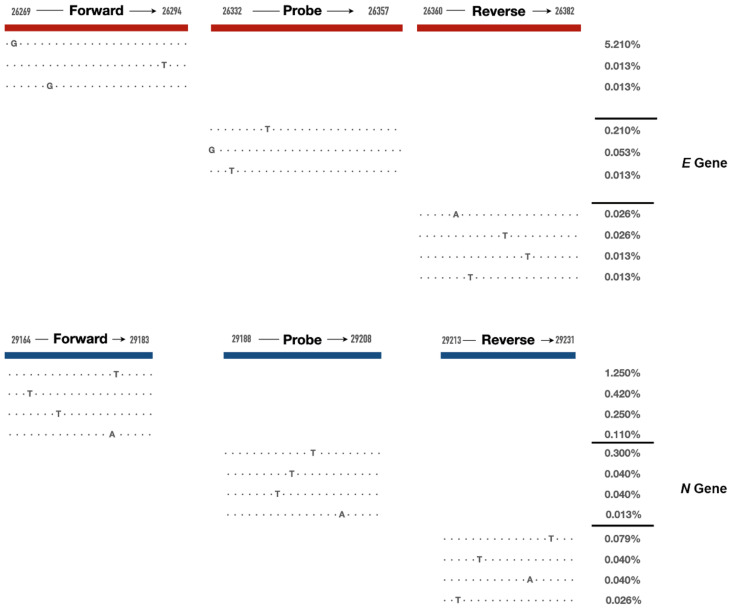
Mutations in the primers and probes identified in the local New York City communities. Wild-type sequences are shown as dots, and sites with mutations at the highest frequency are indicated by the mutated nucleotide. The percentages of genomes with mutations out of all genomes are listed on the right.

We further identified the significant mutations that occurred at a high frequency in the regions of *E* and *N* gene amplicon primers/probes among the isolates from the local New York City communities (Figure 2). The frequency of significant mutations in the viral genome corresponding to the sequences of *E* gene amplicon primers and probe varied from 0.013% to 0.210%. The number of 26340T, 26322G and 26353T mutations that occurred are 16, 4 and 1, respectively, in the region of the *E* gene amplicon probe. The frequency of significant mutations in the viral genome corresponding to the sequences of *N* gene amplicon primers and probe is at a similar level as that in the *E* gene amplicon, ranging from 0.013% to 0.30%. 29200T, 29197T, 29195T, 29204A mutations in the probe region and 29215T in the reverse primer region are significant mutations and likely impact the mRT-PCR detection pattern as reported in the literature. 

Considering this data, we sought to evaluate the utility of our mRT-PCR results on a local level to identify pattern changes in patients with repeat positive testing at least three months from initial infection. We identified 26 unique patients with reinfection. The baseline mRT-PCR results by the Cepheid^®^ Xpert Xpress assay demonstrated three distinct detection patterns: detection of both *N* gene and *E* gene, detection of only *N* gene, and detection of only *E* gene [10]. On retesting, the mRT-PCR detection pattern of viral targets among 26 patients by the same Cepheid^®^ Xpert Xpress assay was found to be different from the pattern seen in their initial infection (Table 1). It is noted that the mRT-PCR detection pattern did not change in the patients on multiple retesting during the clinical course of the initial infection. Similarly, after identifying the change in the detection pattern during retesting, none of the patients were found to have virus variants reflecting the detection pattern from the initial infection.

**Figure 3:**
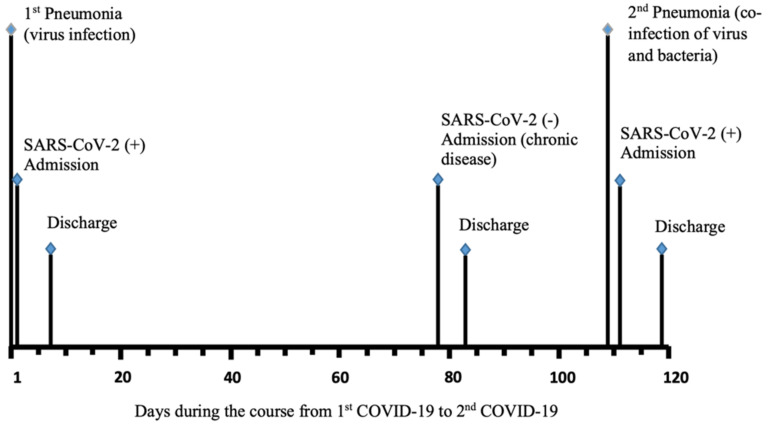
Example of a timeline of reinfected patients’ testing results.

The retesting referenced for each case was conducted either during a readmission or revisit ≥ 3 months (median 150 days) after testing negative with convalescence from the initial infection. In the case of subject 6 (Figure 3), the patient had COVID-19 at the end of April 2020 with the detection of both the *N* gene and *E* gene in a nasopharyngeal swab. Thereafter, the patient tested negative for SARS-CoV-2 RNA in July 2020. However, the patient again had COVID-19 in the middle of August 2021 with a change in the detection pattern to viral *N* gene target-positive only. 

Among the 26 patients, the median age was 67.7 years; 50% were male, 35% white, 23% Asian, 23% Hispanic, and 19% African American. 23/26 (88.5%) had more than three chronic comorbid conditions—most commonly, 73% had hypertension, 35% had diabetes mellitus, and 42.3% had a neurological disorder (e.g., stroke, seizure disorder, cerebral palsy, or dementia). 69.2% of patients were from assisted living facilities. On initial infection, 19/26 (73%) had viral pneumonia. 7/26 (27%) were either persons under investigation for COVID-19 or under surveillance. On subsequent retesting with a change in the detection pattern, 3/26 (11.5%) had viral pneumonia, one of whom died from COVID-19 with multi-organ failure. 5/26 (19.2%) had pneumonia with bacterial superinfection, with two resulting in death. There were four patients with nonspecific symptoms possibly attributable to SARS-CoV-2—1/26 (3.8%) had dyspnea; 3/26 (11.5%) had gastrointestinal complaints (gastritis, diarrhea, or loss of appetite). 8/26 (30.8%) were admitted to be evaluated for other medical problems. 6/26 (23.1%) were persons under investigation for COVID-19 or under surveillance. Overall, 17/26 (65.4%) did not have respiratory symptoms on retesting. Three patients who did not have symptoms of viral pneumonia on initial testing had lower viral loads than in symptomatic patients. 

Among 26 patients, four types of conversions of mRT-PCR detection patterns were observed (Table 1). The mRT-PCR detection pattern changed from the detection of both the *N* gene and *E* gene to that of either the *N* gene (*n* = 16) or *E* gene (*n* = 2) only, from only the *N* gene to both the *N* gene and *E* gene (*n* = 5), and from only the *E* gene to both the *N* gene and *E* gene (*n* = 3). The five specimens testing only *E* gene-positive were re-tested positive by the BioFire COVID-19 assay, which has three different targets to detect viral ORF 1ab and ORF 8 genes. In addition, the SARS-CoV-2 variant with mutations in the *E* gene may have begun circulating in New York City during May 2020 and may have become dominant over the previous variant. The mutations in the genome corresponding to the primers/probe sequences of the *N* gene and the *E* gene amplicon result in changes to the mRT-PCR detection pattern. It would be of interest to further investigate the impact of these mutations on envelope protein and nucleocapsid protein detection. The mutations could lead to changes in the amino acids, protein structure, and antigenicity.

**Figure 4:**
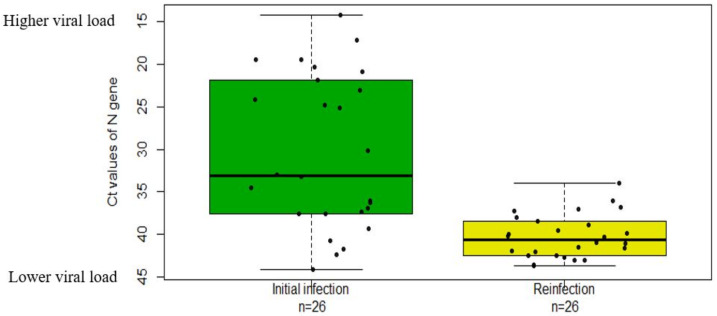
Comparison of mRT-PCR Ct values between the initial infection test and reinfection test. Ct values were generated from the *N* gene amplicon of the Xpert Xpress SARS-CoV-2 assay. For five subjects without *N* gene amplicon detection in either the initial or reinfection test, Ct values from the *E* gene amplicon of both the initial infection and the reinfection were used for analysis. Median values are represented by bolded horizontal lines, minimum and maximum values are represented by non-bolded horizontal lines, and boxes represent the 25–75th percentiles.

We further analyzed the humoral immune responses following SARS-CoV-2 infection and the potential for a subsequent infection. Four of 26 patients tested positive for SARS-CoV-2 antibodies. Our results showed that the viral load in the subsequent infection, measured at a median of 150 days after the initial infection, was significantly lower than that in the previous infection (Figure 4). These results imply that antibodies may play an important role in inhibiting virus amplification during reinfection [11].

## 3. Discussion

The clinical characterization of SARS-CoV-2 variants remains a challenge due to the lack of rapid and cost-effective diagnostic tools, its broad clinical presentation, and its novelty [12]. The fast evolution of the SARS-CoV-2 virus generates genetic changes within a timescale of months. At some time points in New York City, mutations in one gene amplicon were not observed, but mutations were generally continuously seen in two gene amplicons in both New York State and globally. This can largely be attributed to the greater variety of viral variants isolated from the larger number of cases in New York State and worldwide, where the absence of mutations was rarely observed. Recently, we identified that a point mutation, which consisted of up to 5.21% (396/9516 sequenced genomes) in the region of the *E* gene amplicon forward primer, was associated with the outbreak of Omicron at the end of 2021. Determining the mutation frequency and spectrum in viral genomes over time can provide insight into designing a gene amplification-based assay and utilizing the mRT-PCR detection pattern to identify viral variants.

Our data analysis on continuous genetic divergence using next generation sequencing provides explanations for the changes in the mRT-PCR detection pattern after testing negative reflecting differences in viral genomic sequences between variants present in nasopharyngeal swabs collected during the two time points [13]. 

Furthermore, our mRT-PCR evaluation suggests that mRT-PCR with distinct detection patterns on retesting after testing negative from the initial infection may complement a clinical diagnosis in order to assist in identifying virus variants. However, in some instances, virus variants were observed without a documented negative mRT-PCR result even after a significant amount of time had passed between two positive mRT-PCR results for different detection patterns. For example, we had patients with positive mRT-PCR results for different detection patterns six months apart from each other. 

The changes in the mRT-PCR detection patterns of SARS-CoV-2 viral targets on retesting indicate the potential for mRT-PCR to trace SARS-CoV-2 mutant geographic distribution, complement clinical diagnosis, investigate transmission dynamics, and gain insights into prevention and control. This study warrants further investigation on using mRT-PCR with various sets of strain specific primers/probes to identify genotypic changes in SARS-CoV-2 virus variants, which can be evaluated by the Next Generation Sequencing of variants with different detection patterns. 

## 4. Materials and Methods

### 4.1. Sample Collection

Since the start of the COVID-19 pandemic (March 2020), our lab tested 193,054 specimens for SARS-CoV-2 RNA by diagnostic mRT-PCR. To investigate SARS-CoV-2 continuous genetic divergence, we performed Next Generation Sequencing (NGS) on the positive specimens from our lab and LabQ diagnostics with mRT-PCR cycle threshold (Ct) value <33 cycles. We further evaluated all patients with positive repeat SARS-CoV-2 RNA test results between March 2020 and October 2021. Following the guidance issued by the Centers for Disease Control and Prevention, we defined reinfection as any patient with two documented positive test results of ≥3 months apart and a documented negative test in between [14]. An analysis of mRT-PCR by Cepheid^®^ SARS-CoV-2 assay on Infinity was performed to identify any changes in the detection pattern. Lastly, out of those patients who met the definition of repeat positive testing, we analyzed the available SARS-CoV-2 serology data and clinical presentation.

### 4.2. SARS-CoV-2 RNA Detection

SARS-CoV-2 RNA present in nasopharyngeal swab specimens was assayed by mRT-PCR using the Cepheid^®^ Xpert Xpress SARS-CoV-2 assay on Infinity. This assay consists of two amplicons with specific sets of primers/probes. Amplicon 1 targets the region in the viral nucleocapsid gene unique to SARS-CoV-2. Amplicon 2 targets a conserved region of the viral protein envelope gene homologous to all coronaviruses of the Sarbecovirus sub-genus. In addition, a Sample Processing Control and a Probe Check Control are also included for the assay performance. The assay analytical sensitivity was determined by serial dilutions of ZeptoMetrix virus stock—NATSARS(CoV2)-ERC with a known concentration. The limit of detection is 30 virions per assay.

### 4.3. Viral Genomic Amplification and Next Generation Sequencing

Viral RNA is extracted from viral transport medium containing a nasopharyngeal swab, and WGA then starts with the cDNA synthesis of the viral RNA by reverse transcriptase using random hexamer primers. Next, the cDNA of the viral genome is amplified by two separate PCR reactions, whose products are subsequently pooled together. Afterwards, the fragments undergo bead-based tagmentation, where they are tagged to the adapter sequences. Following this, the adapter-tagged fragments undergo another round of PCR amplification; then, using the purification beads, the indexed tagged libraries are pooled and cleaned.

Pooled libraries are clustered onto a flow cell and then sequenced on the NovaSeq 6000 Sequencing System. VarSeq is used for sequence analysis. 7586 isolates out of 9516 sequenced isolates were filtered out by Nextclade-assessed “qc.overallStatus” (v1.11.0, https://github.com/nextstrain/nextclade, assessed on 20 April 2022) as “good” [15] and are subjected to mutation analysis. 

### 4.4. Genomes Collection and Mutation Calling

All the available genomes and their correlated metadata in the Global Initiative on Sharing All Influenza Data (GISAID) collected between 24 December 2019 and 11 February 2022 were downloaded [16,17,18]. The alignment of these sequences was pretreated by GISAID using MAFFT v7.4.90 [19]. Genomes containing less than 27,000 nucleotides of identified bases (A, T, C and G) or without a complete isolation date were removed. Finally, a total of 7,771,134 genomes were collected. Each genome was mapped against the SARS-CoV-2 reference genome (EPI_ISL_402125 in GISAID accession or NC_O45512.2 in GeneBank accession) [20]. For the aligned file, mutations against the reference sequence were called with a homemade Python script.

### 4.5. Mutation Analysis

We analyzed the mutations within the viral genome corresponding to the sequences for *E* gene amplicon primers and probe: 26269-26294 (forward primer), 26360-26381 (reverse primer) and 26332-26358 (probe region), as well as N gene amplicon primers and probe: 29164-29183 (forward primer), 29213-29231 (reverse primer) and 29188-29208 (probe). Each collected genome was checked to see if any mutations occurred in the primers and probe region. All mutations in the primer and probe region were extracted. Any mutations within the probe region and located up to five nucleotides from the 3′ end of primers were classified as significant mutations.

### 4.6. Comparison of Viral Mutations in the Local New York City Communities with Those Globally and in New York State

To investigate the mutation spectrum over time in the local New York City communities, we identified the mutations with our sequenced 7586 genomes. These genomes were divided into different collections by week according to their isolation date. The numbers of collected genomes in each week of a year were calculated. The mutation frequency was calculated by counting all the mutations of the same type and dividing this number by the number of all genomes. The calculated frequencies vs. time were plotted with the matplotlib package in Python. With the same method, we identified mutation spectrums over time in the whole of New York State and globally, and compared the results from the three geographic territories to each other.

## 5. Conclusions

Our data highlight the significance of continuous genetic divergence on the gene amplification-based assay and the value of the mRT-PCR detection pattern in complementing the clinical diagnosis of reinfection. Moreover, our approach, which utilized whole genome amplification and Next Generation Sequencing, can be applied more broadly to identify mutation hotspots throughout the whole genome, which will improve the design of the PCT-based gene amplification assay to empower the gene amplification-based assay in clinical diagnosis.

## Figures and Tables

**Table 1:** Changes in multiplex RT-PCR detection patterns.

Subject No.	Age	Sex	Antibody Positive	Detection Pattern of Initial Infection Test		Detection Pattern of Reinfection Test		Days between Tests
				*E*	*N*	*E*	*N*	
1	69	M	Y	+	+	−	+	133
2	38	F	NT	+	+	−	+	90
3	85	F	NT	+	+	−	+	94
4	63	F	NT	+	+	−	+	100
5	35	F	NT	−	+	+	+	105
6	66	F	NT	+	+	−	+	109
7	51	M	NT	+	+	−	+	116
8	88	F	Y	+	+	−	+	142
9	60	M	NT	+	+	−	+	173
10	84	M	Y	+	+	−	+	155
11	65	F	Y	+	+	−	+	165
12	45	M	NT	+	+	−	+	179
13	86	M	NT	+	+	−	+	183
14	32	F	NT	+	+	−	+	176
15	93	M	NT	+	+	−	+	230
16	47	M	NT	−	+	+	+	186
17	68	M	NT	+	+	+ *	−	223
18	91	M	NT	+	+	−	+	321
19	55	M	NT	−	+	+	+	240
20	52	M	NT	−	+	+	+	312
21	55	M	NT	+ *	−	+	+	98
22	87	F	N	+ *	−	+	+	103
23	81	F	NT	+	+	+*	−	90
24	85	F	NT	−	+	+	+	116
25	84	F	NT	+ *	−	+	+	105
26	76	F	NT	+	+	−	+	227

*E*, envelope; *N*, nucleocapsid; +, detected; −, not detected; Y, yes; N, no; NT, not tested. * These specimens were retested as positive by the BioFire COVID-19 assay.

## Data Availability

The data presented in this study are available upon request from the corresponding authors.
